# Focus on algae and aquatic plants

**DOI:** 10.1093/plphys/kiag650

**Published:** 2026-09-03

**Authors:** Alistair J McCormick, Laura H Gunn, Benedict M Long, Yusuke Matsuda, Douglas J Orr

**Affiliations:** Institute of Molecular Plant Sciences, School of Biological Sciences, and Centre for Engineering Biology, University of Edinburgh, Edinburgh EH9 3BF, United Kingdom; Plant Biology Section, School of Integrative Plant Science, Cornell University, Ithaca, NY 14853, United States; School of Science, University Drive, The University of Newcastle, Callaghan, NSW 2308, Australia; Department of Bioscience, School of Biological and Environmental Sciences, Kwansei Gakuin University, Sanda, Hyogo 669-1330, Japan; Lancaster Environment Centre, Lancaster University, Lancaster LA1 4YQ, United Kingdom

Aquatic photosynthetic organisms carry out roughly half of global net CO_2_ fixation, yet they do so under conditions of variable resource availability quite unlike those experienced by terrestrial plants. For example, CO_2_ diffuses around four orders of magnitude slower in water than in air (dissolved inorganic carbon (C_i_) is predominantly present as HCO_3_^−^ rather than CO_2_), and light, temperature, pH, and nutrient availability can fluctuate on timescales from seconds to seasons. The evolutionary responses to these constraints comprise a host of unique features and characteristics, including biophysical CO_2_-concentrating mechanism (CCM) components, such as carboxysomes and pyrenoids, which represent some of the most striking photosynthetic innovations found in nature, distinctive light-harvesting and photoprotective systems, and a tight integration of photosynthetic and respiratory electron transport.

This Focus Collection brings together updates and topical reviews, and research articles on photosynthetic organisms from aquatic and semiaquatic environments. The contributions span the major photosynthetic lineages, including cyanobacteria, green and red microalgae, diatoms, the hornworts (the only land plants that retain a pyrenoid), and the floating aquatic angiosperm duckweed. Together, they explore the independent, convergent evolution of CCMs across cyanobacteria, algae, and hornworts, and expand the tools available to study these fascinating organisms. Furthermore, the collection discusses a variety of exciting applications, including the development of algae and cyanobacteria as production platforms and engineering strategies to transfer biophysical CCMs into terrestrial crops to enhance photosynthetic performance and yield.

## One challenge, many solutions: CCMs across the aquatic lineages

A recurring theme in this Focus Issue is the remarkable evolutionary convergence of the biophysical CCMs found in prokaryotes and eukaryotes. Pritchard et al. (2026) discuss the pyrenoid-based CCMs (pCCMs) found in algae and hornworts and show that, across an extraordinary diversity of form, three features appear to recur: a condensed Rubisco matrix, specialized membranes that deliver C_i_ to that matrix, and a diffusion barrier that restricts CO_2_ leakage. They contend that comparing the model pCCM from the green alga *Chlamydomonas reinhardtii* with emerging insights from other algae and hornworts could help to distinguish conserved strategies from lineage-specific characteristics. Increasing our understanding of pCCM diversity also provides additional engineering opportunities to assemble pCCMs in vascular plant chloroplasts.

Robison et al. (2026) highlight the hornworts, an enigmatic group of bryophytes that are currently receiving significant focus in the pCCM field. Hornworts are the only land plants with a pyrenoid, and as organisms far closer to crops phylogenetically than any alga, they may help to reveal which pCCM features are retained in the transition to a land plant chloroplast, and whether these can be reintroduced into vascular plants. Shifting from land plants to the marine environment, Matsuda et al. (2026) review the molecular architecture of the diatom pCCM as a series of modules, following initial C_i_ uptake, through translocation to the chloroplast stroma, to controlled release of CO_2_ within the diatom pyrenoid matrix, which is surrounded by a characteristic protein shell that is hypothesized to act as a diffusion barrier to minimize C_i_ leakage and prevent futile cycling, analogous to the starch sheath found in *Chlamydomonas* and other green algae.

Carbonic anhydrase (CA) is a critical enzyme for the function of biophysical CCMs. Moroney (2026) revisits the important roles of CAs in plants, algae and cyanobacteria. The Update also argues against the long-standing assumption about CAs in plants and algae that chloroplast CAs supply CO_2_ to Rubisco in C3 photosynthesis. Arabidopsis mutants lacking chloroplast CA instead show unaltered CO_2_ assimilation, with the essential role of CAs being to supply HCO_3_^−^ to other carboxylases, including the acetyl-CoA carboxylase step of fatty acid biosynthesis. CA function is also shaped by trace-metal availability. Most CAs use a zinc ion as a cofactor, but Avilan et al. (2026) show that *Phaeodactylum tricornutum* expresses a metal-free CA from the newly identified iota class (Pt-ιCA) that is induced under zinc scarcity. Notably, zinc limitation did not prevent CCM induction, which depends on zinc-containing CAs, while Pt-ιCA provides a metal-free route to CA activity that may help sustain C_i_ homeostasis in the zinc-poor marine environments that diatoms often inhabit.

Finally, two contributions address how CCM effectiveness and Rubisco catalysis have coevolved. Iñiguez et al. (2026) compare Rubisco kinetics, photosynthetic CO_2_ affinities and CCM effectiveness across a range of aquatic taxa, including cyanobacteria and algae, and discuss how aquatic phototrophs follow a Rubisco-CCM coevolutionary trajectory distinct from that of terrestrial plants. Aguiló-Nicolau et al. (2026) examine representative taxa spanning the full phylogenetic breadth of the phylum Cyanobacteria. They find diverse Rubisco kinetics overlying a shared evolutionary signature of high catalytic turnover and low CO_2_ affinity, a trade-off permitted by the highly effective carboxysome-based CCM (cCCM). They also report a strong positive correlation between Rubisco's intrinsic carbon isotope fractionation and its CO_2_/O_2_ specificity factor, with implications for using isotopic signatures to reconstruct past atmospheric CO_2_. Bianconi (2026) discusses the broader significance of these findings in an accompanying News and Views.

## Regulation and engineering of the cyanobacterial CCM

Cyanobacteria research articles contribute the largest group to this collection, with two focusing on cCCM-associated C_i_ uptake. Walke et al. (2026) characterize SbtC, an 80-amino-acid protein encoded by a previously unannotated gene located near the *sbtAB* operon in *Synechocystis* sp. PCC 6803. The bicarbonate transporter SbtA is a key cCCM component that is already known to be regulated by the PII-like protein SbtB. Walke et al. show that SbtC is a third partner that interacts with the SbtAB complex and may play a role in modulating SbtA activity and stability, particularly under fluctuating light and C_i_ conditions (see McNelly (2026) for the accompanying News and Views). Regulation of C_i_ uptake is also discussed at the post-translational level by Xin et al. (2026), who identify a new protein (cKAT) as a lysine acetyltransferase critical for regulating the CO_2_ hydration protein ChpX, thus coupling protein acetylation with carbon fixation capacity.

Two further studies focus on the carboxysome. Hu et al. (2026) show that overexpressing β-carboxysomes in *Synechocystis* increases both photosynthesis and growth. This work provides intriguing evidence that carboxysome abundance is not maximized in wild-type cells, and could be engineered for improved performance in biotechnological applications. Rillema et al. (2026) describe a new methodology based on the LI-COR aquatic gas exchange chamber to directly measure CO_2_ assimilation rates by gas exchange—a technique routine in plant physiology but not in aquatic organisms (the latter typically rely on indirect O_2_ evolution measurements). Their approach revealed subtle carboxysome mutant phenotypes not detectable by growth assays, thus offering a potentially more sensitive method to measure aquatic photosynthesis under different controlled conditions.

## Light capture, photoprotection, and the integration of energy metabolism

Aquatic phototrophs experience light regimes that are qualitatively and quantitatively more variable than those on land. The collection reflects a corresponding emphasis on light response and energy balance. Ling et al. (2026) show that cyanobacteria can also optimize light capture through movement, using pilus-mediated phototaxis to reposition relative to a light source. In *Synechocystis*, the lysine methyltransferase Sll0171 methylates the type IV pilus subunit PilA1, and Δ*sll0171* mutants are characterized by increased pilus abundance and sharply enhanced phototactic motility, demonstrating that methylation normally restrains piliation and motility. Sll0171 also methylates key photosynthetic components, such as phycobilisome subunits and ATP synthase, linking phototactic positioning to photosynthetic performance. Wey et al. (2026) provide an update on cyanobacterial electron transport, where photosynthetic, respiratory, and auxiliary pathways share a single thylakoid membrane and compete for a common pool of electron carriers. They also provide a detailed, critical appraisal of the appropriate techniques and associated challenges to measure electron transport in vivo, which often differ from those for plants and algae. Intarasit and Inwongwan (2026) address a related challenge in eukaryotic algae, where photosynthesis and respiration are instead coordinated between separate chloroplast and mitochondrial compartments. They discuss a variety of strategies that have evolved to stabilize chloroplast redox state and buffer productivity under fluctuating light and nutrient conditions, including increased mitochondrial association with the chloroplast in diatoms to enhance malate and oxaloacetate shuttling, and the use of alternative oxidases and cyclic electron flow in green algae and euglenoids.

Focusing on photoprotective mechanisms, Giossi et al. (2025) revisit a long-standing assumption that photoprotection in diatoms relies almost exclusively on the diadinoxanthin cycle, with the violaxanthin cycle (used by most photosynthetic eukaryotes, including land plants) considered a minor contributor. Using *Phaeodactylum tricornutum*, they show that pigments of the violaxanthin cycle can trigger nonphotochemical quenching and that both pathways work synergistically. The pH-dependent regulation of xanthophyll cycling itself depends on a broader ionic environment within the chloroplast. Wunder et al. (2026) show that disrupting KEA-mediated ion homeostasis at the chloroplast envelope in *Chlamydomonas* perturbs plastid rRNA processing and cell cycle progression, with both conserved and lineage-specific consequences. Thus, chloroplast ion homeostasis not only sustains the biochemistry of photosynthesis but also regulates plastome gene expression and, via retrograde signaling, nuclear transcription.

## Metabolic regulation across the diel cycle

Photosynthetic organisms have evolved to carefully balance metabolism across the diel cycle, transitioning from the degradation of stored carbohydrates in the night to the optimization of photosynthetic efficiency in the day. Three studies examine how metabolism is managed across that transition in aquatic phototrophs. Zhang et al. (2026) show that the NAD salvage pathway enzyme NMNAT-C in *Synechococcus elongatus* PCC 7942 sustains NAD^+^ homeostasis specifically during the dark phase, and that NMNAT-C activity is carefully tuned to balance NAD^+^ levels and growth. Hofer et al. (2026) demonstrate the importance of carbon storage in *Synechocystis* by showing that glycogen-deficient Δ*glgC* mutants fail to properly buffer energy and redox balance across the diel cycle. Using single-cell microfluidics, they show that Δ*glgC* mutants have a remarkably narrow margin between growth and light-induced lethality, which underscores their limited physiological flexibility in the absence of a carbon storage buffer. During the day, Δ*glgC* mutants downregulate photosynthesis genes to avoid toxic NADPH accumulation without a carbon sink, and at night they suffer ATP depletion and redox imbalance, leaving cells with an extended lag phase and delayed division after dawn. Lastly, Komiya et al. (2025) establish that target of rapamycin (TOR) signaling in the red alga *Cyanidioschyzon merolae* also controls starch degradation by altering the phosphorylation status of α-glucan water dikinase, thus extending the previously known role of TOR in starch synthesis.

## From aquatic metabolism to bioproduction and bloom control

Microalgae and cyanobacteria are attractive as chassis for bioproduction but can also form blooms that can have negative environmental impacts. Ma et al. (2025) report on how β-carotene, the precursor of the high-value ketocarotenoid astaxanthin, is transported from the chloroplast to the cytoplasm by the budding of plastoglobuli during early astaxanthin hyperaccumulation in *Haematococcus pluvialis*. The latter intracellular trafficking step had not previously been implicated in this system. Transcriptomic analysis further identified a set of candidate genes involved, offering a starting point for investigating the molecular regulation of the hyperaccumulation pathway. Chen et al. (2025) characterize the plastidial glycerol-3-phosphate acyltransferase GPAT1 of *Chlamydomonas* and found that the enzyme also possesses lysophosphatidic acid acyltransferase activity. Knocking down GPAT1 reduced triacylglycerol content but triggered compensatory upregulation of the endoplasmic reticulum localized GPAT2. This substantially increased production of 1,3-olein-2-palmitin, a functional lipid used in infant formula, and positions GPAT1 as a target for engineering lipid composition in microalgae. Bloom control is addressed by Enkerlin et al. (2026), who used a metabolomic profiling approach to investigate the toxicity of lysine to cyanobacteria. Using the model *Synechocystis*, the authors traced the response to disruption of multiple metabolic pathways, including amino acid turnover, peptidoglycan biosynthesis and photosynthesis. To identify the genetic basis of lysine resistance, the authors employed a CRISPRi-based genetic screen and identified several targets, including the basic amino acid and glutamine transporter (Bgt) system, providing insights into potential regulatory pathways and avenues for future research in controlling cyanobacterial blooms.

## Enabling technologies to study aquatic phototrophs

Expanding research beyond model species is a key goal to develop a broader, more global mechanistic understanding of biological processes. Nam et al. (2026) present a modular Golden Gate toolkit for the centric diatom *Thalassiosira pseudonana* in which a single episome delivered by bacterial conjugation supports a variety of genetic modification tools, including scarless endogenous tagging (eg with fluorescent proteins) and CRISPR-Cas9 editing. The authors demonstrated the utility of the kit by knocking out Diatom Pyrenoid Component 1 while simultaneously GFP-tagging the Rubisco small subunit (RbcS). Furthermore, they endogenously tagged the bestrophin-like protein BST2 and confirmed its localization to the pyrenoid under low CO_2_, supporting a role in diatom pCCM. Uranga (2026) discusses the significance of this novel toolkit for diatom genome engineering in an accompanying News and Views. Ruiz-Sola et al. (2026) take a complementary approach in the model *Chlamydomonas* by establishing the *chlL* mutant, which lacks a functional dark-operative chlorophyll synthesis pathway, as a model for time resolved studies of light-induced chloroplast biogenesis. Dark adapted *chlL* cells accumulate a de-differentiated, chlorophyll free plastid, but can rebuild a functional chloroplast from starch reserves within 1–3 hours of illumination. The authors further show that lincomycin, an inhibitor of chloroplast translation, blocks light-induced biogenesis and triggers retrograde signaling to the nucleus, establishing lincomycin as a tool for studying plastid to nucleus communication.

Russo et al. (2025) discuss the optimization of shotgun proteomics workflows to analyze cyanobacterial proteomes. Using the model *Synechococcus elongatus* PCC 7942, the authors demonstrate that improved sample preparation methods paired with library-free data-independent acquisition mass spectrometry allow for detection of over 80% of the predicted proteome, achieving the greatest single-shot coverage yet for a cyanobacterium. Finally, Rintoul et al. (2026) outline a suite of methodologies for the LI-COR aquatic gas exchange chamber (used by Rillema et al. (2026) for cyanobacteria) to measure CO_2_ assimilation and electron transport in duckweed. Duckweeds are among the fastest-growing floating macrophytes and are increasingly attractive for both fundamental photosynthesis research and applied production. By combining gas exchange and chlorophyll fluorescence measurements the authors compare the photosynthetic performance of different duckweed species. Furthermore, the recent development of duckweed transformation protocols raises the possibility of genetically engineering enhanced photosynthetic traits, which could be screened using these gas exchange methods.

## Data Availability

No new data were generated or analysed in support of this research.
